# Phylum *Verrucomicrobia *representatives share a compartmentalized cell plan with members of bacterial phylum *Planctomycetes*

**DOI:** 10.1186/1471-2180-9-5

**Published:** 2009-01-08

**Authors:** Kuo-Chang Lee, Richard I Webb, Peter H Janssen, Parveen Sangwan, Tony Romeo, James T Staley, John A Fuerst

**Affiliations:** 1 School of Chemistry and Molecular Biosciences, University of Queensland, Brisbane, Queensland 4072, Australia; 2 Centre for Microscopy and Microanalysis, University of Queensland, Brisbane, Queensland 4072, Australia; 3 AgResearch Limited, Grasslands Research Centre, Tennent Drive, Private Bag 11008, Palmerston North 4442, New Zealand; 4 CSIRO Manufacturing and Materials Technology, Private Bag 33, Clayton South Victoria 3169, Australia; 5 University of Sydney, Sydney, New South Wales, Australia; 6 Department of Microbiology, University of Washington, Seattle, WA 98195, USA

## Abstract

**Background:**

The phylum *Verrucomicrobia *is a divergent phylum within domain Bacteria including members of the microbial communities of soil and fresh and marine waters; recently extremely acidophilic members from hot springs have been found to oxidize methane. At least one genus, *Prosthecobacter*, includes species with genes homologous to those encoding eukaryotic tubulins. A significant superphylum relationship of *Verrucomicrobia *with members of phylum *Planctomycetes *possessing a unique compartmentalized cell plan, and members of the phylum *Chlamydiae *including human pathogens with a complex intracellular life cycle, has been proposed. Based on the postulated superphylum relationship, we hypothesized that members of the two separate phyla *Planctomycetes *and *Verrucomicrobia *might share a similar ultrastructure plan differing from classical prokaryote organization.

**Results:**

The ultrastructure of cells of four members of phylum *Verrucomicrobia *– *Verrucomicrobium spinosum*, *Prosthecobacter dejongeii*, *Chthoniobacter flavus*, and strain Ellin514 – was examined using electron microscopy incorporating high-pressure freezing and cryosubstitution. These four members of phylum *Verrucomicrobia*, representing 3 class-level subdivisions within the phylum, were found to possess a compartmentalized cell plan analogous to that found in phylum *Planctomycetes*. Like all planctomycetes investigated, they possess a major pirellulosome compartment containing a condensed nucleoid and ribosomes surrounded by an intracytoplasmic membrane (ICM), as well as a ribosome-free paryphoplasm compartment between the ICM and cytoplasmic membrane.

**Conclusion:**

A unique compartmentalized cell plan so far found among Domain Bacteria only within phylum *Planctomycetes*, and challenging our concept of prokaryote cell plans, has now been found in a second phylum of the Domain Bacteria, in members of phylum *Verrucomicrobia*. The planctomycete cell plan thus occurs in at least two distinct phyla of the Bacteria, phyla which have been suggested from other evidence to be related phylogenetically in the proposed PVC (*Planctomycetes-Verrucomicrobia-Chlamydiae*) superphylum. This planctomycete cell plan is present in at least 3 of 6 subdivisions of *Verrucomicrobia*, suggesting that the common ancestor of the verrucomicrobial phylum was also compartmentalized and possessed such a plan. The presence of this compartmentalized cell plan in both phylum *Planctomycetes *and phylum *Verrucomicrobia *suggest that the last common ancestor of these phyla was also compartmentalized.

## Background

The phylum *Verrucomicrobia *forms a distinct phylogenetically divergent phylum within the domain Bacteria, characterized by members widely distributed in soil and aquatic habitats. Cells of some species such as *Verrucomicrobium spinosum *and *Prosthecobacter dejongeii *possess cellular extensions termed prosthecae and cells of other strains occur in an ultramicrobacteria size range [1,2]. Verrucomicrobia are significant for our understanding of both bacterial evolution and microbial ecology. At present, six monophyletic subdivisions (subphyla, classes) are recognized within the phylum *Verrucomicrobia* on the basis of 16S rRNA gene library studies [3,4]. There are more than 500 different verrucomicrobia 16S rRNA gene sequences in publicly-accessible databases, but only a handful of these represent cultivated strains. The verrucomicrobia pose interesting evolutionary questions – members of at least one genus, *Prosthecobacter*, possess genes for a homolog of eukaryotic tubulin, unknown in other prokaryotes, along with the bacterial tubulin-like protein FtsZ. *Verrucomicrobium spinosum *possesses a FtsZ divergent from those in other phyla of the domain Bacteria [5-8]. In addition, some members of the verrucomicrobia have been recently found to oxidize methane and use methane as a sole source of carbon and energy, making them the only known aerobic methanotrophs outside the proteobacteria, and the only extreme acidophilic methanotrophs known [9-11]. They are thus significant for our understanding of the evolution of methanotrophy and C1 transfer biochemistry.

It has recently been proposed that the phyla *Planctomycetes*, *Verrucomicrobia *and *Chlamydiae *of the domain Bacteria form a superphylum called the PVC superphylum, which may also include the phyla *Poribacteria *and *Lentisphaerae*. The *Planctomycetes*, *Chlamydiae Verrucomicrobia*/*Lentisphaerae *grouping is supported by 16S and 23S rRNA sequence analysis [12,13]. Another study based on both phylogenetics of concatenated protein datasets and shared conserved inserts in proteins has supported the link between the phyla *Verrucomicrobia *and *Chlamydiae *[14]. Other studies based on either 16S and 23S rRNA gene sequences [15], or individual or concatenated protein sequences [16,17], have shown no specific relationships between the three phyla, *Verrucomicrobia*, *Planctomycetes *and *Chlamydiae*. However, for one of these studies [15] sequences from some superphylum lineages were not yet available and thus sequence selection may have influenced tree topology. In another of these studies [17], the inability to detect the PVC superphylum may have resulted from a loss of resolution due to editing concatenated sequence data to allow inclusion of a wide range of taxa including those of Eukaryotes. It is known that all members of the phylum *Planctomycetes *so far examined possess a characteristic cell plan involving compartmentalization of the cell cytoplasm by an intracytoplasmic membrane (ICM) separating the cytoplasm into two regions, the inner ribosome-containing pirellulosome and the less central ribosome-free paryphoplasm [18,19]. The term "pirellulosome" was first introduced to describe a major nucleoid-containing cell compartment of planctomycetes bounded by an internal membrane, the intracytoplasmic membrane (ICM). A ribosome-free "paryphoplasm" region surrounds the pirellulosome and is separated from it by the ICM [18]. Based on the proposed relationships between the three lineages, we hypothesized that members of *Planctomycetes *and *Verrucomicrobia *might share a similar ultrastructure plan. This is investigated in this study using transmission electron microscopy incorporating techniques such as high pressure freezing, cryosubstitution and freeze fracture, to examine four verrucomicrobia representing three of the six subdivisions.

## Results

By applying high-pressure freezing, cryosubstitution and freeze-fracture techniques, internal compartmentalization of the cell has been observed in four representatives of the phylum *Verrucomicrobia*. The four species examined, *Verrucomicrobium spinosum*, *Prosthecobacter dejongeii*, *Chthoniobacter flavus*, and verrucomicrobia strain Ellin514, represent four genera and three distinct subdivisions (1, 2 and 3) of the phylum. Cells of all four species were examined after high-pressure freezing and cryosubstitution or after preparation of replicas of freeze-fractured cells. Cells of all four displayed features that are consistent with compartmentalization of the cell cytoplasm by internal membranes.

### Cell compartmentalization in *Verrucomicrobium spinosum*

**Figure 1:**
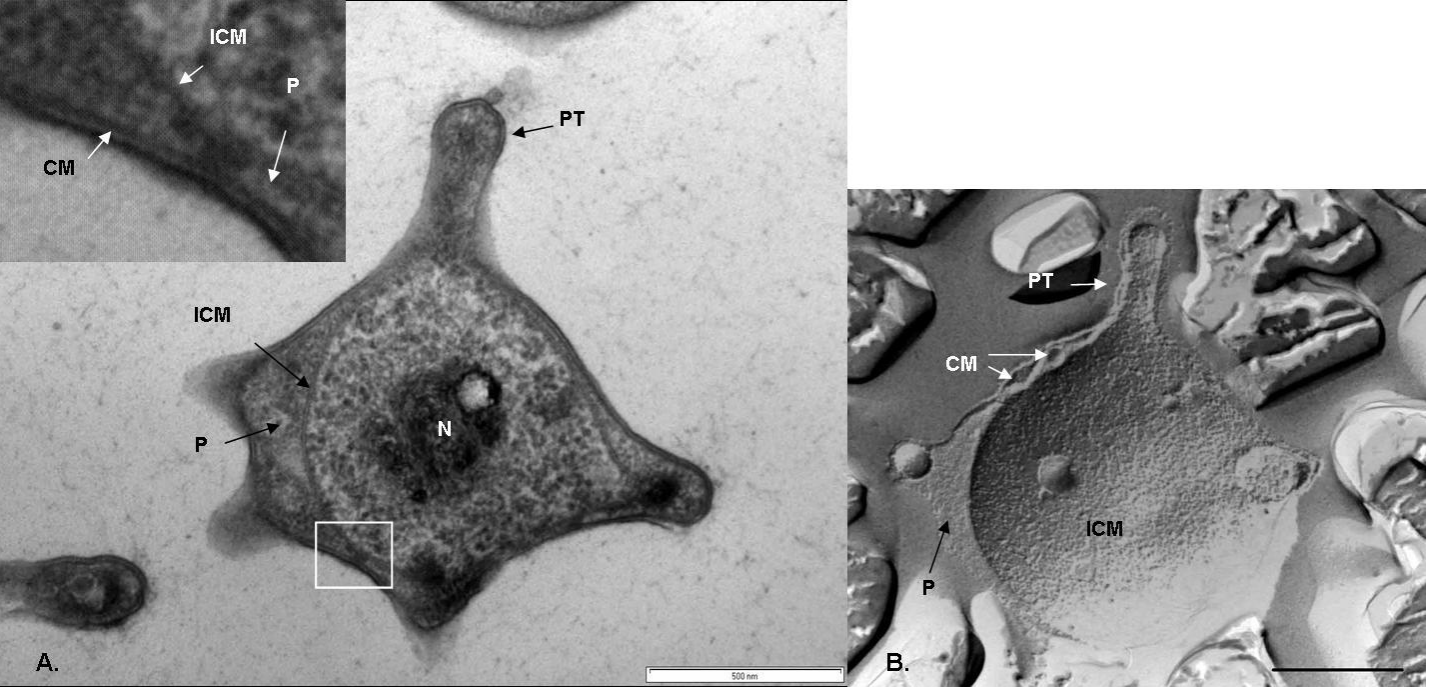
**Transmission electron micrographs of high-pressure frozen and cryosubstituted *Verrucomicrobium spinosum***. A. Cell prepared by high-pressure freezing and cryosubstitution showing prostheca (PT), paryphoplasm (P), and an intracytoplasmic membrane (ICM) enclosing a pirellulosome region containing a condensed fibrillar nucleoid (N). Inset: enlarged view of area of cell outlined in the white box showing cytoplasmic membrane (CM), paryphoplasm and ICM. B. freeze-fracture replica of cell showing cross-fractured paryphoplasm (P) and fracture faces of ICM and CM. Bar – 500 nm

**Figure 2:**
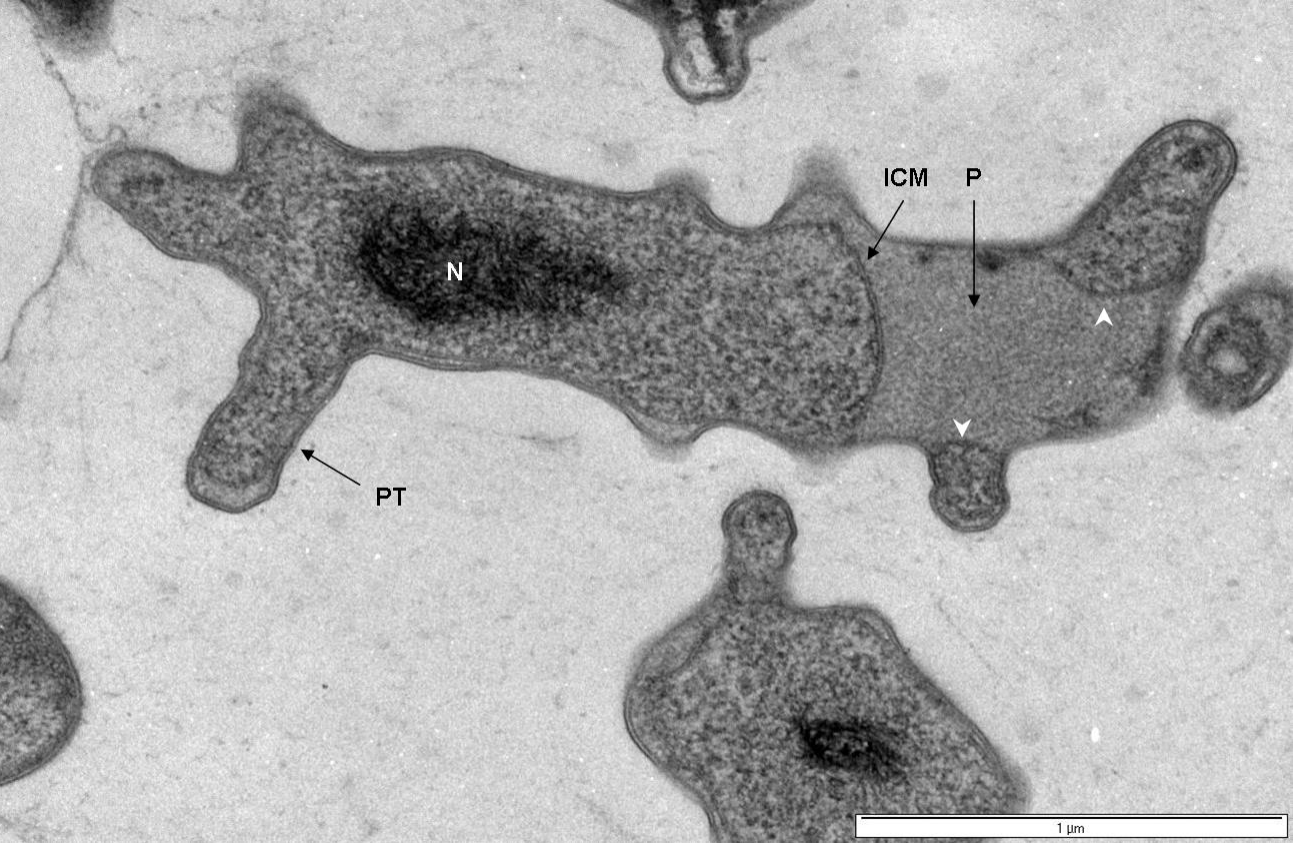
**Transmission electron micrograph of high-pressure frozen and cryosubstituted *Verrucomicrobium spinosum***. Cell prepared by high-pressure freezing and cryosubstitution showing prostheca (PT), ribosome-free paryphoplasm (P), and an intracytoplasmic membrane (ICM) enclosing a pirellulosome region containing a condensed fibrillar nucleoid (N). Membrane-bounded vesicle-like compartments within some prosthecae extensions are also present (see arrowheads). Bar – 1 μm

Internal compartmentalization was observed in thin-sectioned cells prepared via high-pressure freezing and cryosubstitution (Fig. 1A, 2). Classical features of a typical bacterium are clearly visible in cells of *Verrucomicrobium spinosum*, such as a nucleoid, cytoplasmic membrane (CM) and a cell wall. However, an internal membrane surrounds a region containing the nucleoid and ribosome-like particles, which thus forms a membrane-bounded compartment similar to the planctomycete pirellulosome. This internal membrane has the typical trilaminar structure of a classic bilayer unit membrane seen via electron microscopy of thin-sectioned cells, i.e., two dense layers on either sides of an electron-transparent layer. The mean membrane width (7.0 nm ± 1.1 S.D.) is consistent with that typical for unit membranes [20]. This pirellulosome-like compartment in *V. spinosum *is filled with particles with an electron density and diameter consistent with the classical characteristics of ribosomes and is surrounded by a ribosome-free region (i.e., with no electron-dense particles of characteristic diameter and shape) equivalent to the paryphoplasm cell compartment of planctomycetes [18]. In most cells, the paryphoplasm is markedly different in texture and electron density to the cytoplasm in the pirellulosome (Fig. 2). In addition to the major pirellulosome compartment containing the nucleoid, there are also apparently separate smaller membrane-bounded vesicle-like compartments in some cells (Fig. 2), often seen within the prosthecal extensions. These do not contain nucleoid, but are filled with ribosome-like particles. The texture of the small compartments and the pirellulosome cytoplasm are similar and this texture differs from that of the paryphoplasm. These small membrane-bounded compartments outside the nucleoid-containing pirellulosome may represent extensions of the main pirellulosome, since the cell is only viewed in two-dimensional section.

In addition to the compartmentalization by an internal membrane, *Verrucomicrobium spinosum *also contains a condensed fibrillar nucleoid, confined within a localized region of the pirellulosome. The distinctive multiple prosthecae of *Verrucomicrobium spinosum *can also be seen (Fig. 1A).

Examination of a freeze-fracture replica of *Verrucomicrobium spinosum *clearly confirms the presence of a major intracytoplasmic membrane (ICM) seen in a fracture along its surface and the presence of a paryphoplasm external to this ICM (Fig. 1B). Freeze-fracture also clearly confirms the presence of the cytoplasmic membrane, which is seen in fracture along its surface as distinct from the surface-fractured ICM and separated from it by the cross-fractured paryphoplasm (Fig. 1B).

**Figure 3:**
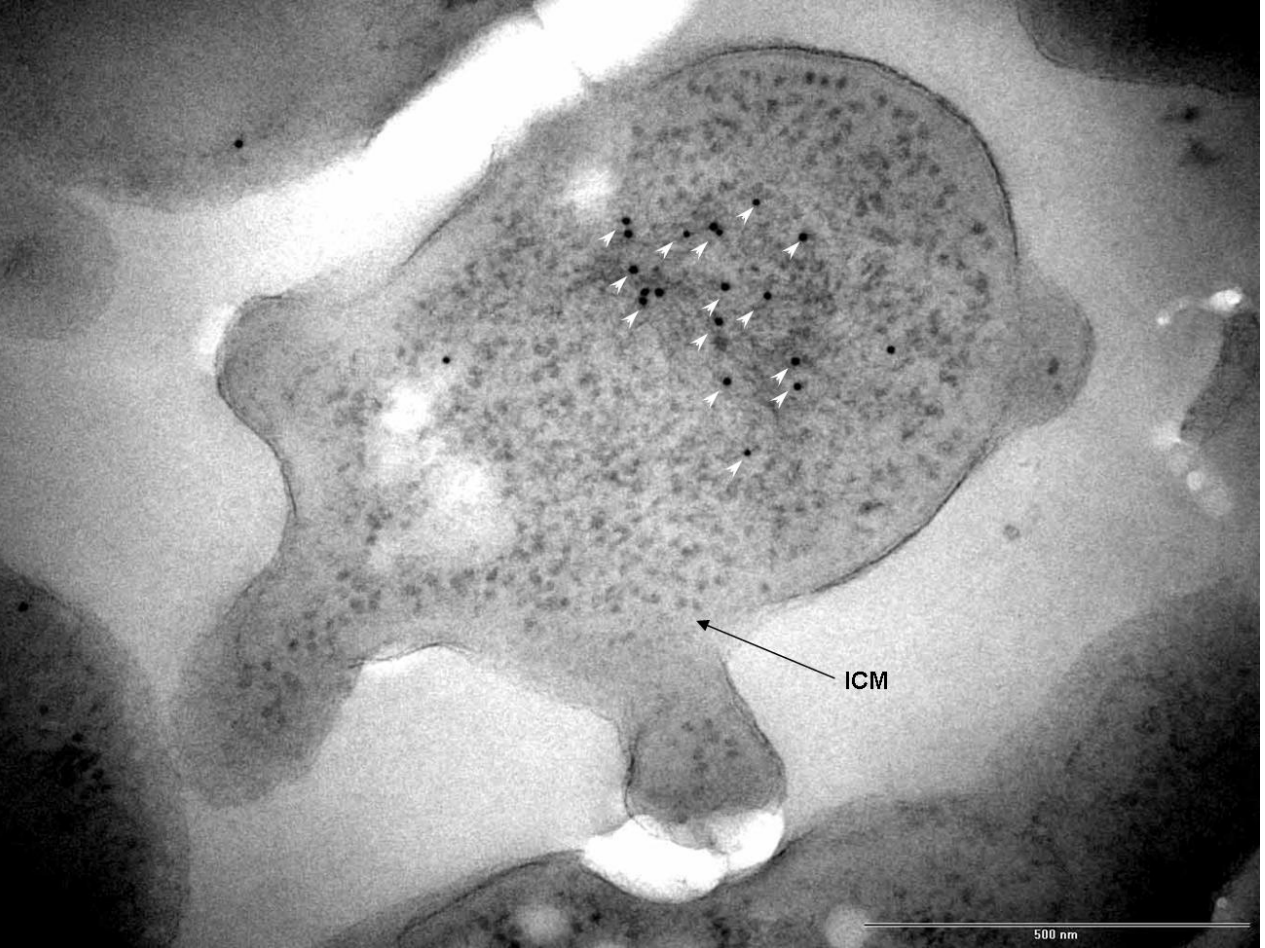
**Transmission electron micrograph of high-pressure frozen and cryosubstituted cell of *Verrucomicrobium spinosum*, immunogold labelled using anti-double-stranded DNA mouse monoclonal antibody and goat anti-mouse IgG bound to 10-nm-colloidal gold, showing labelling only over the condensed fibrillar nucleoid (white arrowheads) which is contained within a pirellulosome bounded by an intracytoplasmic membrane (ICM)**. Bar – 500 nm.

Immunogold labeling for double-stranded DNA shows most of the cell DNA, as expected, is within the dense fibrillar nucleoid located in the major membrane-bounded pirellulosome compartment, as indicated by a high number of gold particles deposited in this region (Fig. 3). Due to the absence of osmium tetroxide during cryosubstitution, the paryphoplasm is unstained and relatively electron-transparent in these cells.

### Cell compartmentalization in *Prosthecobacter dejongeii*

**Figure 4:**
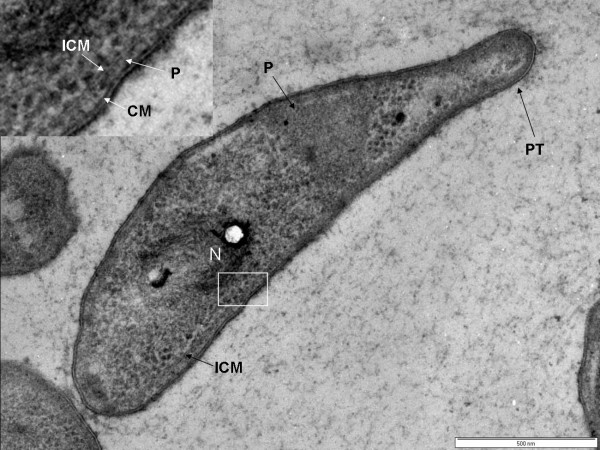
**Transmission electron micrograph of high-pressure frozen and cryosubstituted cell of *Prosthecobacter dejongeii*, showing prostheca (PT), an intracytoplasmic membrane (ICM) surrounding a pirellulosome region containing a condensed fibrillar nucleoid (N), and a paryphoplasm region (P)**. Inset: enlarged view of region of cell outlined in the white box showing cytoplasmic membrane (CM), paryphoplasm (P) and ICM. Bar – 500 nm.

**Figure 5:**
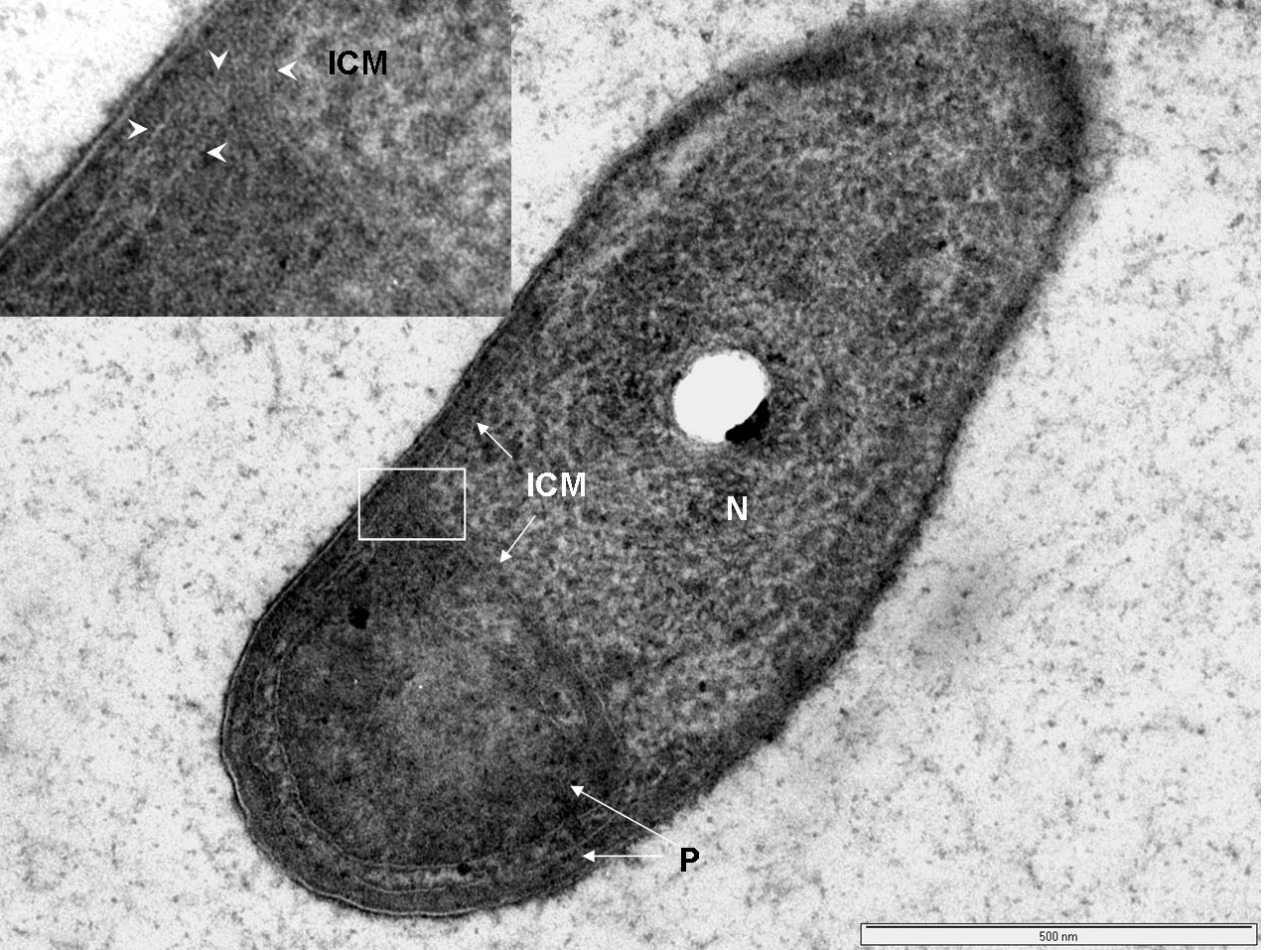
**Transmission electron micrograph of high-pressure frozen and cryosubstituted cell of *Prosthecobacter dejongeii *showing an intracytoplasmic membrane (ICM) surrounding a pirellulosome region containing a fibrillar nucleoid (N), paryphoplasm region at cell rim and a large invagination of rim paryphoplasm (P) at the cell pole**. Inset: enlarged view of region of cell periphery showing continuity of the paryphoplasm at the cell rim with a large polar invagination of paryphoplasm, which is bounded by ICM which also defines an extension of the pirellulosome's riboplasm into the cell pole (see arrowheads). Bar – 500 nm.

*Prosthecobacter dejongeii *also shares the basic cell plan possessed by the *Planctomycetes*. A typical prosthecobacter cell shape and a distinctive prostheca can be easily recognized in Fig. 4. High-pressure frozen and cryosubstituted preparations of cells of *Prosthecobacter dejongeii *also revealed internal compartmentalization consisting of a major single membrane-bounded region containing the fibrillar nucleoid and all the ribosome-like particles of the cell (Figs 4, 5). An ICM with a mean width of 5.0 nm ± 0.5 S.D. surrounds and defines this nucleoid- and ribosome-containing region. In some cells there appeared to be more than one of these membrane-bounded compartments, but closer examination revealed a connection between the compartments, which thus appear to represent one major membrane-bounded compartment rather than separate compartments (Fig. 4). Other regions of the cell were apparently ribosome-free and formed a cell compartment in between the ICM and the cytoplasmic membrane and cell wall. This compartment is equivalent to the paryphoplasm of planctomycetes, and in *Prosthecobacter *cells appears to surround the cell rim but also can occur as regions extending from the cell rim through the cell centre (Fig. 4 and Fig. 5). These appear to separate membrane-bounded ribosome-containing regions in cells where several of these appear to occur (Fig. 4 and 5). However, narrow extensions of ribosome-containing cytoplasm seem to connect such superficially separate membrane-bounded regions, suggesting there is only one major membrane-bounded ribosome and nucleoid-containing organelle. The complexity of the way in which the ICM can enclose the membrane-bounded ribosome-containing region within the ribosome-free paryphoplasm (that is, the way in which the paryphoplasm can surround the ICM) is illustrated in Fig. 5, where there is a large invagination of paryphoplasm at one cell pole and where continuity of this region with the outer rim of paryphoplasm is apparent. Thus, the underlying topology of the cell plan in *Prosthecobacter *is that of a large ribosome- and nucleoid-containing compartment equivalent to the planctomycete pirellulosome, bounded by a single ICM membrane separating that compartment from a ribosome-free paryphoplasm.

**Figure 6:**
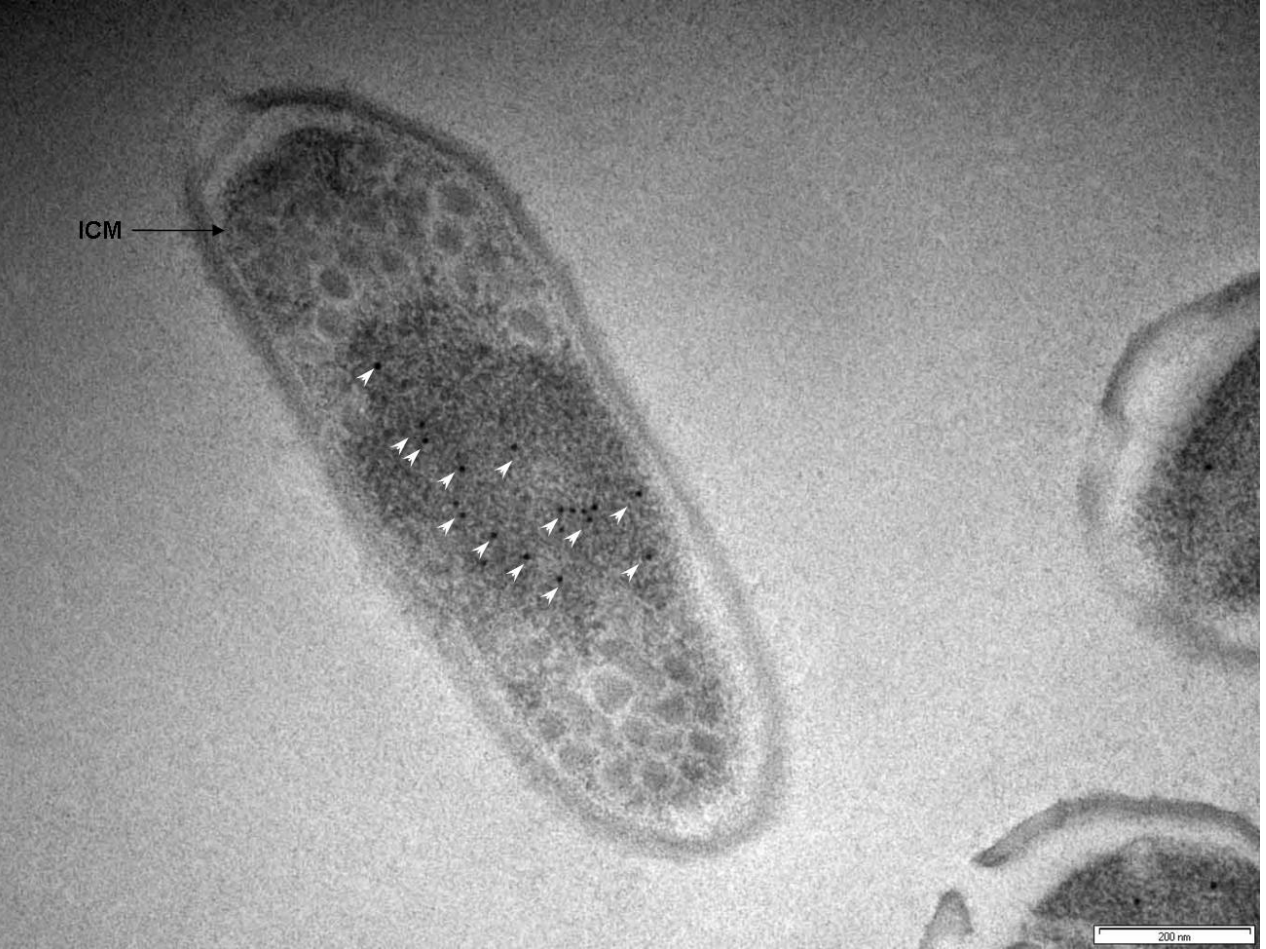
**Transmission electron micrograph of high-pressure frozen and cryosubstituted cell of *Prosthecobacter dejongeii*, immunogold labeled using anti-double-stranded DNA mouse monoclonal antibody and goat anti-mouse IgG bound to 10 nm-colloidal gold, showing labeling only over the condensed fibrillar nucleoid (white arrowheads) in the pirellulosome bounded by an intracytoplasmic membrane (ICM)**. Bar – 200 nm.

Immunogold labeling of double-stranded DNA shows that the DNA is, as expected, coincident with the dense fibrillar nucleoid located within the major membrane-bounded compartment of the cell (Fig. 6).

### Cell compartmentalization in *Chthoniobacter flavus*

**Figure 7:**
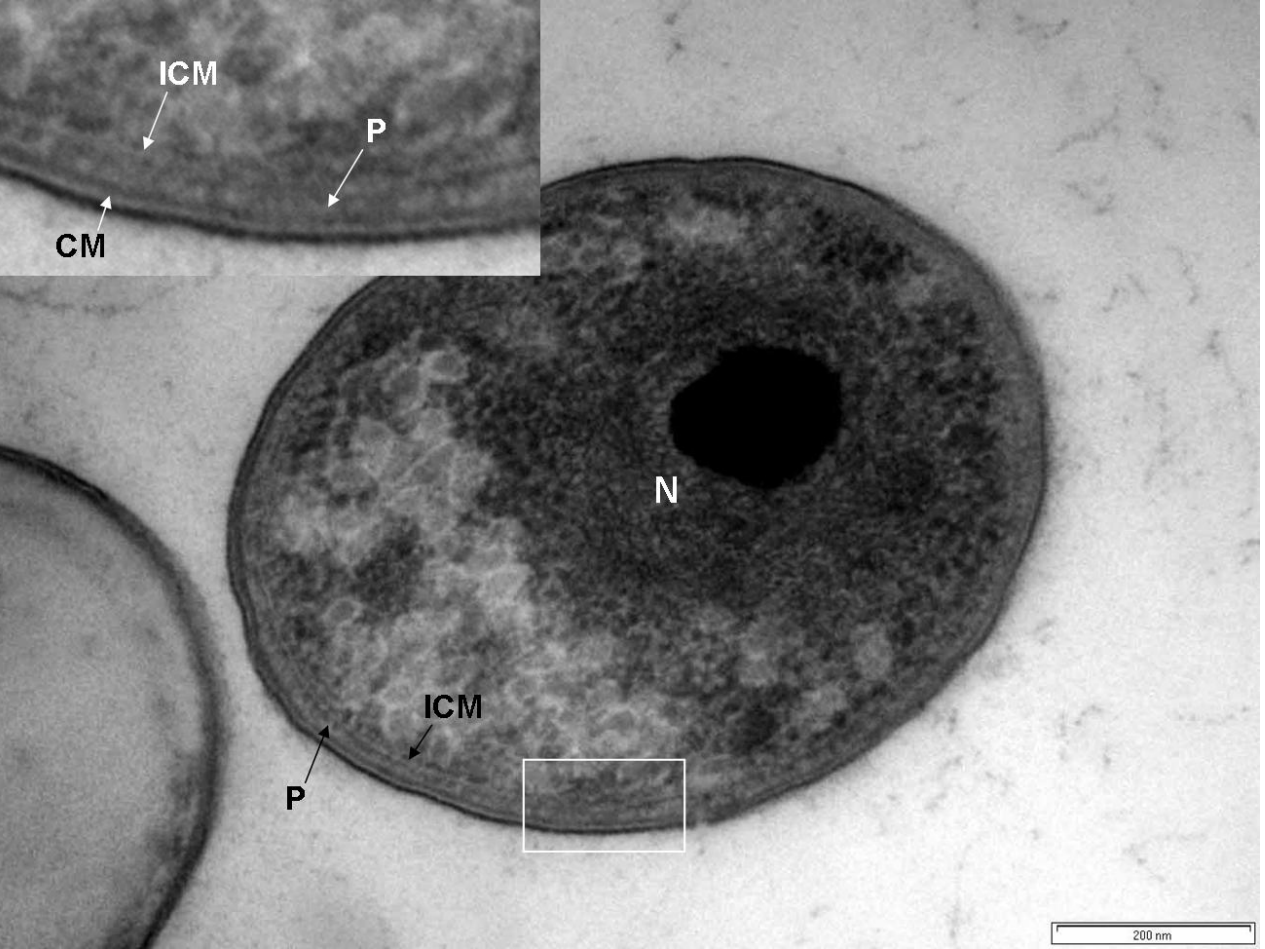
**Transmission electron micrograph of high-pressure frozen and cryosubstituted cell of *Chthoniobacter flavus*, showing paryphoplasm (P) and an intracytoplasmic membrane (ICM) enclosing a pirellulosome region containing a condensed fibrillar nucleoid (N) which surrounds an electron-dense granule**. Inset – enlarged view of region of cell outlined in the white box showing cytoplasmic membrane (CM), paryphoplasm (P) and intracytoplasmic membrane (ICM). Bar – 200 nm.

In high-pressure frozen and cryosubstituted *Chthoniobacter flavus*, as in *V. spinosum *and *P. dejongeii*, cells were found to possess two major compartments separated by a membrane analogous to those characteristic of the planctomycete cell plan. The larger compartment, equivalent to a planctomycete pirellulosome bounded by the ICM, contains a condensed fibrillar nucleoid that can enclose large electron-dense granule-like structures (Fig. 7). The smaller paryphoplasm-equivalent compartment surrounds the pirellulosome and lies between the ICM and the CM.

### Cell compartmentalization in strain Ellin514

**Figure 8:**
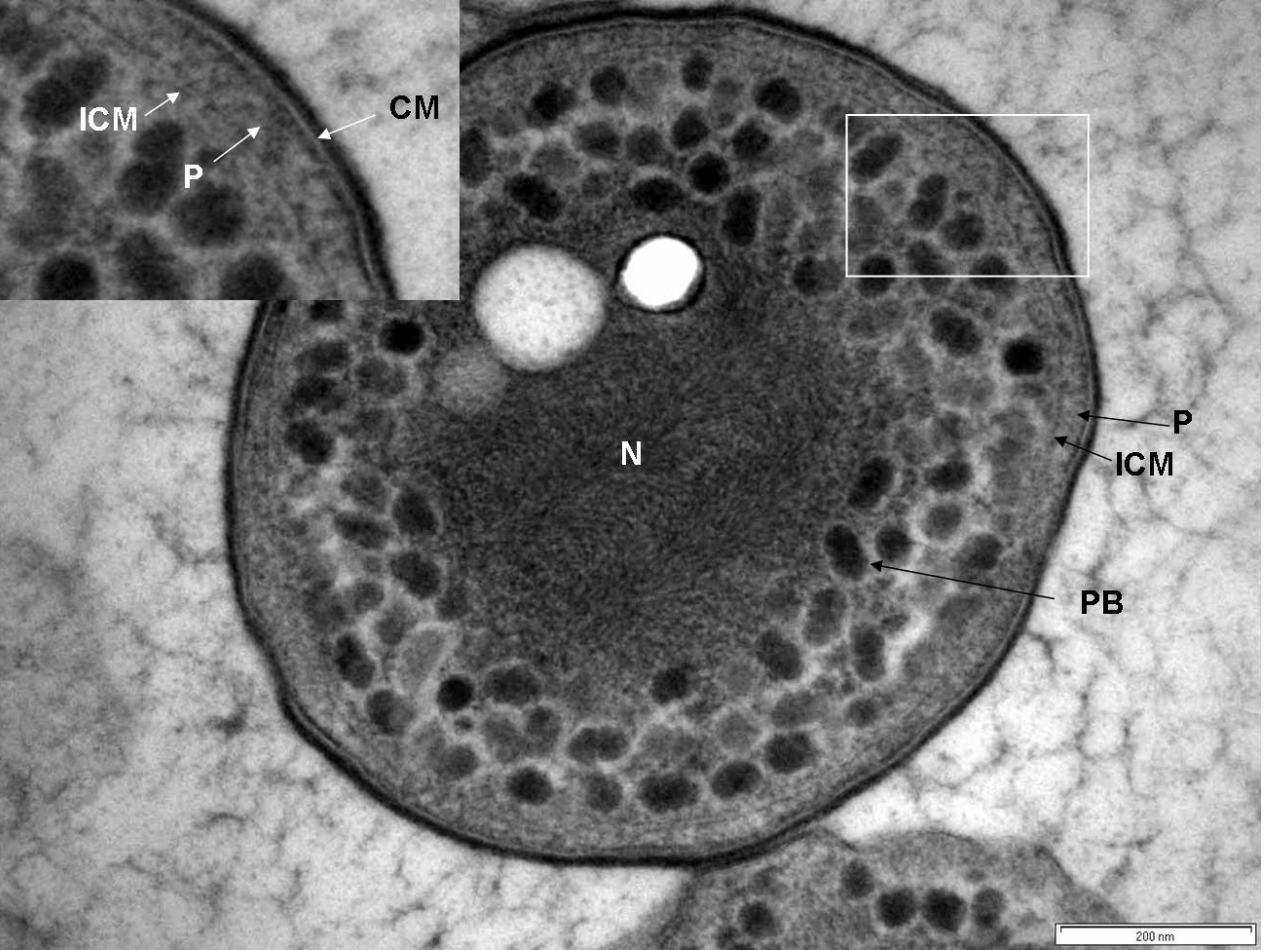
**Transmission electron micrograph of high-pressure frozen and cryosubstituted cell of verrucomicrobia strain Ellin514, showing paryphoplasm (P), and intracytoplasmic membrane (ICM) enclosing a pirellulosome possessing polyhedral bodies (PB) surrounding a condensed fibrillar nucleoid (N) containing granules**. Inset: enlarged view of region of cell outlined in the white box showing cytoplasmic membrane (CM), paryphoplasm (P) and intracytoplasmic membrane (ICM). Bar – 200 nm.

In high-pressure frozen and cryosubstituted strain Ellin514, known to be a representative of subdivision 3 of the phylum *Verrucomicrobia*, cells were also found to possess a major pirellulosome compartment separated by an ICM from an outer paryphoplasm, again analogous to the planctomycete cell plan (Fig. 8). The pirellulosome compartment possessed a condensed fibrillar nucleoid associated with electron-transparent oval granules, and was filled with polyhedral bodies of varying electron density. Ribosomes were not clearly visible and the polyhedral bodies seem to occupy most of the pirellulosome.

## Discussion

We have demonstrated that all four members of the phylum *Verrucomicrobia *examined, *Verrucomicrobium spinosum*, *Prosthecobacter dejongeii*, *Chthoniobacter flavus*, and verrucomicrobia strain Ellin514, share a basic cell plan analogous to that found in members of the phylum *Planctomycetes*. This cell plan is characterized by compartmentalization of the cell cytoplasm by a major cell organelle bounded by a single membrane containing all the cell DNA in a fibrillar condensed nucleoid, as well as ribosome-like particles. This major membrane-bounded organelle is equivalent to the pirellulosome of planctomycetes, and its bounding membrane is equivalent to the intracytoplasmic membrane (ICM) defined in planctomycetes as surrounding the pirellulosome [18]. Consistent with the structural analogies between verrucomicrobia and planctomycetes, the ribosome-free region between the ICM of the pirellulosome and the cytoplasmic membrane in verrucomicrobia can be considered equivalent to the paryphoplasm of planctomycetes. The verrucomicrobial cell plan is most similar to the simplest planctomycete cell plan seen in *Pirellula staleyi*, *Blastopirellula marina *[18] and *Rhodopirellula baltica*. There is no indication of a single membrane-bounded organelle not containing a nucleoid such as the anammoxosome of anaerobic ammonium-oxidizing bacteria, a group thought to represent some of the most deep-branching *Planctomycetes *or even a separate phylum-level lineage within the PVC superphylum [21,22] and which share a cell plan including the pirellulosome with planctomycetes [23-25]. However, the small membrane-bounded regions of ribosome-containing pirellulosome cytoplasm within paryphoplasm in *V. spinosum *resemble features of a pirellula-like planctomycete cultured from a Mediterranean sponge [26]. The cell plan determined in verrucomicrobia was revealed using a cryosubstitution method for preparation of cells before thin-sectioning for electron microscopy, a method comparable to those used previously for establishing the planctomycete cell plan [18,27].

Cells of all the species of verrucomicrobia examined here using high-pressure freezing followed by cryosubstitution also possess condensed nucleoids, which is another feature of similarity to the ultrastructure of planctomycetes. All planctomycetes appear to possess condensed nucleoids when cryofixed cryosubstituted cells are examined [18]. Cryosubstitution, unlike conventional chemical fixation, is not expected to yield such condensation as an artifact of fixation [28-30]. This contrasts with the appearance of nucleoids in cryofixed cells of other bacterial species such as *Escherichia coli *and *Bacillus subtilis*, where a 'coralline' nucleoid extending through the cell cytoplasm is found [28,29]. Chromatin-like nucleoids have been reported in "*Candidatus *Xiphinematobacter", symbionts of nematodes belonging subdivision 2 of *Verrucomicrobia *[4], and also in epixenosome symbionts belonging to subdivision 4 [31], although in both cases these were examined only using chemical fixation. The condensed nucleoids of all the species examined here often contained granules of varying electron density. Such granules within nucleoids have been noted to occur within cryo-fixed cells of *Deinococcus radiodurans *vitreous sections examined by cryoelectron microscopy [32].

*V. spinosum *and *P. dejongeii *are members of subdivision 1 (class *Verrucomicrobiae*) of the phylum *Verrucomicrobia *[1]. There is another member of the phylum *Verrucomicrobia*, *Rubritalea squalenifaciens*, isolated from the marine sponge *Halichondria okadai *and belonging to subdivision 1 *Verrucomicrobia*, which seems to possess the planctomycete-like cell plan in an accompanying published figure, but this interpretation was not made by the authors [33]. The planctomycete cell plan has also been observed in symbiont bacteria studied directly in sponge tissue [34]. Some of those from the sponge *Haliclona caerulea* include cells with multiple prosthecae and in which both ICM and riboplasm were recognized [35]. These bacteria may be verrucomicrobia or prosthecate alphaproteobacteria, with the ultrastructure suggesting the former. At least 3 species of verrucomicrobial subdivision 1 thus appear to possess the planctomycete cell plan. *C. flavus *is a member of subdivision 2 (class *Spartobacteria*) [36], and Ellin514 is a member of subdivision 3 [37] so that we have determined the planctomycete cell plan to be present in at least 3 distinct subdivisions of the phylum *Verrucomicrobia*. This cell plan may occur widely among distinct subdivisions of the phylum *Verrucomicrobia*, which could suggest that the common ancestor of the verrucomicrobial phylum was also compartmentalized and possessed such a plan. The planctomycete cell plan thus occurs in at least two distinct phyla of the Bacteria. These phyla have been suggested to be related phylogenetically in the so-called PVC superphylum [12,38]. Members of the phylum *Poribacteria*, also postulated to belong to the PVC superphylum, have been proposed to be compartmentalized [38], and our electron microscopy examination of thin sections of cells of *Lentisphaera araneosa*, prepared via high-pressure freezing (unpublished data), indicates that at least one member of the phylum *Lentisphaerae *within the PVC superphylum [39] also possesses compartmentalized cells with the planctomycete plan. This plan seems to be shared by members of the PVC superphylum, and it is possible that a common compartmentalized ancestor of the superphylum may have shared the planctomycete cell plan. Other proposed members of the superphylum, such as members of the phylum *Chlamydiae*, should also be examined for such a cell plan. Interestingly, *Parachlamydia acanthamoeba*, a chlamydial organism which occurs as an endosymbiont of free-living amoebae, possesses one stage of its life cycle, the crescent body, which seems to display internal membranes and a cell plan in thin sections consistent with verrucomicrobial and planctomycete plans [40], but this needs to be confirmed using cryo-fixation preparative methods.

Chemically fixed cells of extremely acidophilic methanotrophic members of the phylum *Verrucomicrobia *forming a new subdivision within the phylum have been reported to possess unusual internal structures, including polyhedral bodies and tubular membranes, when thin sections are viewed by transmission electron microscopy [9,10]. It is not possible from those micrographs to deduce any clear relationship of these structures to a planctomycete cell plan, but it is possible that when these strains are prepared by high-pressure freezing they will also be shown to possess such a plan. The internal membrane structures seen sometimes in cells of the methanotrophic verrucomicrobial strain V4 have been suggested to house particulate methane monooxygenase enzymes, as in other known methanotrophs. However, the occurrence of intracytoplasmic membrane similar to those of planctomycetes and in the chemoheterotrophically-grown verrucomicrobia strains studied here suggest that verrucomicrobial internal membranes need not always be associated with a particular metabolism.

The structure of 'epixenosome' verrucomicrobia symbionts of the ciliate *Euplotidium*, members of subdivision 4 of verrucomicrobia, is complex and there has been no suggestion of compartmentalization by internal membranes. However, these cells have so far only been examined by chemical fixation [31]. The structure of the cells of these organisms should be re-examined via cryo-fixation based techniques to determine their consistency with the model proposed here for the verrucomicrobial cell plan, since it is possible that the complex structures found may be accompanied by internal membranes when methods more suitable for their preservation are used.

## Conclusion

A unique cell plan so far found only within the phylum *Planctomycetes *of the Domain Bacteria, and which challenges our concept of the prokaryote cell plan, has now been found in a second bacterial phylum – phylum *Verrucomicrobia*. The planctomycete cell plan thus occurs in at least two distinct phyla of the Bacteria, phyla which have been suggested from other evidence to be related phylogenetically as members of the proposed PVC superphylum. This planctomycete cell plan is present in at least 3 of the 6 subdivisions of the *Verrucomicrobia*, suggesting that the common ancestor of the verrucomicrobial phylum was also compartmentalized and possessed such a plan. The presence of this compartmentalized cell plan in both phylum *Planctomycetes *and phylum *Verrucomicrobia *suggests that the last common ancestor of these phyla was also compartmentalized. Cell compartmentalization of this type may thus have significant meaning phylogenetically, and can act as a clue to the meaning of deeper evolutionary relationships between bacterial phyla. Its occurrence in a second phylum of domain Bacteria extends and reinforces the challenge to the concept of prokaryotic organization already posed by planctomycete cell organization. Definitions of the prokaryote depending on absence of membrane-bounded organelles may require further reexamination, a process already underway [41-43]. Such compartmentalized cell plans may have phylogenetic and evolutionary significance of relevance to such problems as the origin of cell compartmentalization in eukaryotes and the origin of the eukaryotic nucleus. In summary, the cell plan shared by all members of the phylum *Planctomycetes *so far examined appears also to be shared by several members of the phylum *Verrucomicrobia*, suggesting that such a plan may be common to these distinct bacterial phyla, and that the common ancestor of these relatively closely related phyla may have also possessed this plan.

## Methods

### Bacteria and culture conditions

*Verrucomicrobium spinosum *was grown on MMB medium [44] and incubated aerobically at 28°C. *Prosthecobacter dejongeii *and *Chthoniobacter flavus *were grown on DM agar medium [45] both incubated aerobically at 28°C. Strain Ellin514 was grown in VL55 broth medium and incubated aerobically at 28°C [46].

### High-pressure freezing and cryosubstitution

Bacteria cultures were high-pressure frozen with liquid nitrogen using a BalTec HPM-010 or a Leica EMPACT 2 high-pressure freezer. The frozen samples were kept and stored in a 2-ml tube containing liquid nitrogen before cryosubstitution was carried out.

The frozen sample was transferred to a microfuge tube containing 2% (wt/vol) osmium tetroxide in acetone and cryosubstituted in a Leica AFS. The sample was warmed from -160°C to -85°C over 1.9 h (rate 40°C/h), held at -85°C for 36 h, then warmed from -85°C to 20°C over 11 h (4°C/h). The high-pressure frozen and cryosubstituted samples were then processed into EPON resin and ultrathin-sectioned using a Leica Ultracut Ultramicrotome UC61. The cut sections were placed onto a formvar-coated copper grid and stained with 5% (wt/vol) uranyl acetate in 50% ethanol and with lead citrate.

### Freeze fracture

*Verrucomicrobium spinosum *cells were swabbed off a plate and resuspended in 20% (vol/vol) glycerol for 1 hr. After rapid freezing, cells were freeze-fractured using a Balzers BAF 300 Unit. Fracturing was performed at -120°C, and 3 nm of platinum/carbon was shadowed onto the samples at an angle of 45°. A 25 nm layer of carbon was then evaporated on top of this. Samples were taken from the freeze fracture unit and thawed. The replicas were cleaned in 25% chromic acid for 3 days, rinsed 3 times in distilled water and picked up onto 200 mesh copper grids.

### Immunolabelling of double-stranded DNA

Ultrathin-sections of high-pressure frozen and cryosubstituted *V. spinosum *and *P. dejongeii *cells on carbon-coated copper grids were floated onto drops of Block solution containing 0.2% (wt/vol) fish skin gelatin, 0.2% (wt/vol) BSA, 200 mM glycine and 1 × PBS on a sheet of Parafilm, and treated for 1 min at 150 W in a Biowave microwave oven. The grids were then transferred onto 8 μl of primary antibody, (mouse monoclonal IgG anti-double-stranded DNA (abcam) diluted 1:500 in Block solution), and treated in the microwave at 150 W, for 2 min with microwave on, 2 min off, and 2 min on. The grids were then washed on drops of Block solution 3 times, and treated each time for 1 min in the microwave at 150 W, before being placed on 8 μl of goat anti-mouse IgG 10 nm-colloidal gold antibody (ProSciTech) diluted 1:50 in Block solution and treated in the microwave at 150 W, for 2 min with microwave on, 2 min off, and 2 min on. Grids were washed 3 times in 1 × PBS, each time being treated for 1 min each in the microwave at 150 W, and 4 times in water for 1 min each in the microwave at 150 W. The grids were dried and stained with 1% (wt/vol) aqueous uranyl acetate. Three negative controls were carried out for this experiment. Firstly, anti-GFP antibody, an antibody which targeted an antigen not expected to occur in *Verrucomicrobia*, was used as the primary antibody. Secondly, the block solution with no antibody of any type was used in place of the primary antibody. Thirdly, sections were treated with DNase before the labeling procedures. Two replicates per species were performed for the immunogold labeling experiment.

### Transmission electron microscopy

All high-pressure frozen and cryosubstituted sections and freeze-fracture replicas were viewed using a JEOL 1010 transmission electron microscope operated at 80 kV. Images were captured using iTEM 5.0 universal TEM image platform software. The resulting files were annotated and resolution adjusted for final image production using Photoshop CS.

## Authors' contributions

K-CL cultured and prepared cells for high-pressure freezing and electron microscopy, and performed electron microscopy. RIW assisted K-CL with expert knowledge of high-pressure freezing cell preparation. TR performed freeze-fracture and production of fracture replicas. PS isolated pure cultures of Ellin strains of verrucomicrobias and shared drafting the manuscript. PHJ supplied pure cultures of Ellin strains and contributed expert knowledge of phylum Verrucomicrobia phylogenetics. JTS supplied pure cultures of *Verrucomicrobium spinosum *and *Prosthecobacter dejongeii *and contributed expert knowledge of phylum *Verrucomicrobia*. K-CL and JAF wrote the manuscript and RIW, PS, PHJ and JTS contributed to drafting the manuscript. JAF conceived of the study, participated in its design and coordination and helped to write the manuscript. All authors read and approved the final manuscript.
